# Lactobacillus improves meat quality in Sunit sheep by affecting mitochondrial biogenesis through the AMPK pathway

**DOI:** 10.3389/fnut.2022.1030485

**Published:** 2022-10-28

**Authors:** Chenlei Wang, Xinlei Yan, Yanping Bai, Lina Sun, Lihua Zhao, Ye Jin, Lin Su

**Affiliations:** College of Food Science and Engineering, Inner Mongolia Agricultural University, Hohhot, China

**Keywords:** Lactobacillus, myofiber, mitochondrial biogenesis, AMPK pathway, meat quality

## Abstract

Sunit sheep are famous for their high meat quality, but the meat quality of them has declined due to the change in feeding methods. Lactobacillus has a variety of probiotic effects and is widely used in animal diets to optimize meat quality. This study aimed to investigate the effect of dietary supplementation with different levels of Lactobacillus on meat quality. A total of 24 3-month-old Sunit sheep with an average body weight of 19.03 ± 3.67 kg were randomly divided into control (C), 1% (L1), 2% (L2), and 3% Lactobacillus groups (L3), with 6 sheep in each group. Myofiber characteristics, meat quality, and metabolic enzyme activity were detected. Moreover, the regulatory mechanism of Lactobacillus on meat quality was explored by using Western blotting and real-time Quantitative polymerase chain reaction (RT-qPCR). The results showed that dietary addition of Lactobacillus decreased LDH activity in the Biceps femoris of Sunit sheep (*P* < 0.05). Compared to the other groups, the 1% Lactobacillus group showed the conversion of myofibers from the glycolytic to the oxidative type, and the increasing b* values (*P* < 0.05), decreasing shear force and cooking loss of meat (*P* < 0.05) and the relative gene and protein expression levels of AMPK, PGC-1α, NRF1, TFAM, and COX IV (*P* < 0.05) in the Biceps femoris were also increased in the 1% Lactobacillus group. Therefore, the addition of Lactobacillus to the diet of Sunit sheep could regulate the AMPK signaling pathway to promote myofiber type conversion, which improves meat quality. This study provided a theoretical and data basis for improving the meat quality of sheep and supplied a novel way of applying Lactobacillus.

## Introduction

Sunit sheep is a special breed of grassland sheep in Inner Mongolia, China under long-term natural selection and using the traditional natural grazing method; the breed model is popular among consumers for its good meat quality and high nutritional value (1). In recent years, the feeding method of Sunit sheep has altered to grazing from barn feeding for the development of large-scale farming and the implementation of the national policy of pasture protection, which had decreased its meat quality (2). Therefore, improvement of the meat quality of Sunit sheep is vital and urgent. Kang et al. (3) found that the deposition of intramuscular fat could improve the tenderness and water retention of meat. Nemati et al. (4) discovered that vitamin E increased the antioxidant capacity of meat, which improved meat quality. It has also been shown that meat quality can be optimized by modulating the transformation of myofiber types (5).

Myofibers are the basic constituent unit of animal muscle that can be classified into four types according to the polymorphism of myosin heavy chain (MyHC): slow oxidizing type (I), fast oxidizing type (IIa), fast glycolytic type (IIb) and intermediate type (IIx) (6). And according to ATPase staining, they can be classified into three types: type I, type IIA and type IIB. The metabolic enzyme activity levels and structural protein contents of different myofibers are different and have diverse effects on meat quality (7). It has been found that type I myofibers play a positive role in meat quality (8). Meat with a high content of type I myofibers has higher water retention and tenderness and a brighter red color, and vice versa for meat with type II myofibers. It was reported that myofiber types could be converted as follows: I ↔ IIa ↔ IIx ↔ IIb, which were affected by breed, feeding model, sex, age and nutrition (9–13). Therefore, adjusting myofiber type transformation is an important way to improve meat quality.

Mitochondrial dysfunction could reduce the proportion of slow muscle fiber and increase the proportion of fast muscle fiber in skeletal muscle, providing evidence for the involvement of mitochondria in the transformation of myofiber types (14). Chen et al. (15) discovered that *Lactobacillus paracasei* PS23 maintained mitochondrial function and improved muscle mass in aging mice, providing a certain basis for Lactobacillus-induced mitochondrial biogenesis to transform myofiber types. Moreover, it was reported that lactate could regulate mitochondrial biogenesis and increase the quantity and quality of mitochondria by upregulating AMPK (AMP-activated protein kinase), PGC-1α (peroxisome proliferator-activated receptor gamma coactivator −1α) and other factors (16).

Lactobacillus is a kind of probiotic with an extremely wide range of applications, such as its use as a nutritional additive in animal diets, which can improve the growth performance and immunity of animals and can maintain their intestinal microecological balance (17–20). A recent study revealed that supplementation of Lactobacillus in the diet could increase the proportion of oxidized myofibers and improve the quality of meat in sheep (21). However, little information is available on the mechanism of this phenomenon, especially the role of mitochondrial biogenesis.

In this study, Lactobacillus was added to the diet of Sunit sheep, and myofiber characteristics were tested. Furthermore, the gene and protein expression levels of factors related to the AMPK signaling pathway that can regulate mitochondrial biogenesis in muscle were determined to uncover the mechanism by which Lactobacillus affects the transformation of myofiber types. This study provided not only a new method for the application of Lactobacillus but also a technical reference for the production of high-quality sheep meat.

## Materials and methods

### Animals, diet and sample collection

Our animal protocols were approved by the Experimental Animal Welfare Ethics Committee of Inner Mongolia Agricultural University (No: NND2021072). This experiment was conducted in Chuanjing Sapomu, Urat Middle Banner, Bayannur City, Inner Mongolia, China. A total of 24 healthy, 3-month-old purebred Sunit sheep with an average body weight of 19.03 ± 3.67 kg were randomly divided into a control group (C), a 1% Lactobacillus group (L1), a 2% Lactobacillus group (L2) and a 3% Lactobacillus group (L3), with 6 sheep in each group, half male and half female. The control group was fed a basal diet (mainly corn and concentrate feed), which did not contain any antibiotics. The composition and nutritional level of the basal diet were shown in Table 1. The basal diet of the test groups was supplemented with 1, 2, and 3% Lactobacillus (Ruanbang, *Lactobacillus casei* HM-09, *Lactobacillus plantarum* HM-10, 1.5 × 10^9^ cfu/g), respectively. All sheep were individually reared in pens, which were fed once a day and allowed to freely drink water during the experiment. The sheep were killed after 90 days, and then Biceps femoris samples from carcasses were collected for meat quality determination. 100 mg of sample were quickly frozen in liquid nitrogen and stored at –80^°^C for the determination of gene and protein expression and enzyme activity. The Biceps femoris samples were cut into approximately 1 × 0.5 × 0.5 cm^3^ (L × W × H) muscle masses along the direction of myofiber, dehydrated with isopentane, quickly frozen with liquid nitrogen, and stored at –80^°^C for the study of myofiber characteristics.

**TABLE 1:** Basic diet composition and nutritional levels (%).

Raw material composition	Content/%	Nutritional indicators	Content/%
Maize	60.00	Crude protein	18.00
Sunflower cake (meal)	18.00	Crude fiber	11.60
Soybean meal (cake)	13.45	Crude ash	7.80
Vegetable (cotton) seed meal	4.00	Calcium	1.21
Stone powder	1.00	Phosphorus	0.63
Calcium hydrogen phosphate	0.50	Lysine	0.48
Sodium chloride	0.75		
Lysine	0.80		
Vitamin and mineral premix	1.50		
Total	100.00		

Vitamin and mineral premix includes vitamin A, vitamin D_3_, vitamin E, vitamin K_3_, vitamin B_12_, ferrous sulfate, zinc sulfate, copper sulfate, manganese sulfate, etc.

### Determination of meat quality

The pH_0_ (after 45 min of slaughter) and pH_24h_ value (after 24 h (4^°^C) of acid excretion) of the biceps muscle were obtained directly by a pH meter (pH-STAR, Matthaus, Germany), which was calibrated at 4^°^C using standard buffer solution (pH 4.6 and 7.0). For each sample, three measurements were recorded to calculate the average value. Lightness (L*), redness (a*) and yellowness (b*) values of Biceps femoris samples were measured with a chromometer (TC-P2A, Shanghai, China) using a mean of three random readings after a 1 h of blooming time; the chromometer was calibrated with a standardized white tile, at 2^°^C observer angle, 50 mm aperture size, and the illuminant D65. After 24 h of carcass adaptation to 4^°^C, Biceps femoris muscle was removed to measure the cooking loss and shear force. Cooking loss was determined as described by Yang et al. (22). Briefly, each of Biceps femoris sample was weighed (m1/g) and individually placed in a polyethylene bag, and then heated in a 75^°^C water bath (HH-8, Jiangsu, China) until the inner temperature reached 70^°^C; the surface of samples were dried and their post-boiling mass (m2/g) were recorded after recovery to room temperature. Shear force was measured on cooked samples (from cooking loss). The muscle sample was cut into 3 × 1 × 1 cm^3^ (L × W × H) dimensions and then analyzed in parallel to the longitudinal orientation of the muscle fiber with a Digital Meat Tenderness Instrument (C-LM3B, Beijing, China) with parallel five times and the average value was taken.

Cooking loss (%) = (m1 – m2)/m1 × 100%

m1 —the mass of Biceps femoris sample before boiling (g);
m2 —the mass of Biceps femoris sample after boiling (g).

### Determination of myofiber characteristics

Transverse sections (10 μm thickness) were made with a cryostat microtome (Slee, Germany) at –25°C from the entire block of frozen meat sample and stained with ATPase according to Bakhsh et al. (23). A microscope (Leica, Germany) was used to observe and select a clear field of view for slicing (total number of myofibers per sample > 1000). The number, diameter and cross-sectional area of the different types of myofibers were statistically analyzed using Leica Qwin V3 fiber color analysis software.

### Determination of metabolic enzyme activity

The activities of succinate dehydrogenase (SDH), malate dehydrogenase (MDH) and lactate dehydrogenase (LDH) were measured with a commercial kit (Nanjing Jiancheng Bioengineering Institute, China) according to the instructions. Each sample was measured three times in parallel.

### Real-time quantitative polymerase chain reaction

Gene mRNA expression was quantified using real-time quantitative polymerase chain reaction (RT-qPCR) analysis. Total RNA was extracted from muscle using TRIzol Reagent (TaKaRa, Dalian, China), and cDNA was synthesized according to the instruction manual of the PrimeScriptTM RT Reagent Kit with gDNA Eraser (TaKaRa, Dalian, China). The endogenous control was glyceraldehyde-3-phosphate dehydrogenase (GAPDH). The primer sequences used were presented in Table 2. The 2^–ΔΔ*Ct*^ method was used to quantify gene expression levels.

**TABLE 2:** Primers used for RT-PCR.

Gene	Primer sequence (5′-3′)	Product size/bp	Gene bank number
GAPDH	F: CTCAAGGGCATTCTAGGCTACACT	180	NM_001190390.1
	R: GACCATGAGGTCCACCACCCTGT		
MyHC I	F: AAGAACCTGCTGCGGCTG	250	AB058898
	R: CCAAGATGTGGCACGGCT		
MyHC IIa	F: GAGGAACAATCCAATACAAATCTATCT	173	AB058896
	R: CCCATAGCATCAGGACACGA		
MyHC IIb	F: GACAACTCCTCTCGCTTTGG	247	XM_027974883.1
	R: GGACTGTGATCTCCCCTTGA		
MyHC IIx	F: GGAGGAACAATCCAATGTCAAC	178	AB058897
	R: GTCACTTTTTAGCATTTGGATGAGTTA		
AMPKα1	F: TCCGAAGTATTGATGATGA	154	XM_027980096.1
	R: ACAGATGAGGTAAGAGAAG		
NRF1	F: GTCGCTCATCCAGGTTGGTA	118	NM_001100708.1
	R: CACCTCTCCATCAGCCACAG		
PGC-1α	F: GGTGACCATGACTATTGTCAG	105	XM_004009738
	R: CTCGGATTTCCTGGTCTTGAA		
TFAM	F: CGCTCCCCCTTTAGTTTTGC	216	XM_015104510.1
	R: CTGCCAGTCTGCCCTGTAAG		
COX IV	F: CCCATCCCGCACACCTTT	237	XM_012133553.1
	R: CCTCACTTCTTCCACTCGTTCTTG		

### Western blotting

Proteins were extracted from Biceps femoris muscle using lysate (Aladdin, Shanghai, China). The protein concentration was detected using a BCA protein concentration assay kit (Beyotime Biotechnology, Shanghai, China). The proteins were electrophoresed, transferred, immunoblotted, and visualized as described by Xu et al. (24). Primary antibodies against β-actin, and HRP-labeled sheep anti-rabbit (1: 50000, Wuhan Bode, cat. No. BA1054) were n (1: 1000, Wuhan Bode, cat. No. BM0627), AMPK (1: 1000, Cell Signaling, cat. No. 2532), p-AMPK (1: 1000, Cell Signaling, cat. No. 2535), PGC-1α (1: 2000, Abcam Affinity, cat. No. ab106814), NRF1 (recombinant nuclear respiratory factor 1) (1:2000, Affinity, cat. No. AF5298), TFAM (mitochondrial transcription factor A) (1: 1000, Affinity, cat. No. AF0531), and COX IV (Cytochrome C oxidase IV) (1: 1000, Abcam, cat. No. ab33985) and the secondary antibodies HRP-labeled sheep anti-mouse (1: 50000, Wuhan Bode, cat. No. BA1051), HRP-labeled rabbit anti-sheep (1: 50000, Wuhan Bode, cat. No. BA1060) used to detect protein expression.

### Statistical analysis

All text data were analyzed by ANOVA using GLM pro-cedures of SPSS 17.0 software (SPSS Inc., Chicago, IL) and were reported as means and pooled SEM. *P* < 0.05 was considered a significant difference to apply Duncan’s significant difference test.

## Results

### Effects of the dietary addition of Lactobacillus on the meat quality of Sunit sheep

The effects of dietary supplementation with Lactobacillus on the meat quality of Sunit sheep were shown in Table 3. The pH_0_ values were not significantly different among any of the groups (*P*> 0.05). The L* and a* values of the samples were not significantly different among the groups (*P* > 0.05), and the b* value of the L1 group was significantly higher than that of the C group (*P* < 0.05). Compared with the C group, the shear forces of the L2 and L3 groups were significantly increased (*P*< 0.05), but those of the L1 group were significantly decreased (*P* < 0.05). The cooking loss levels in all Lactobacillus groups were significantly lower than that in the C group (*P* < 0.05). This demonstrated that Lactobacillus was able to increase the water retention and tenderness of meat.

**TABLE 3:** Effects of lactobacillus on the meat quality of Sunit sheep.

Item	C	L1	L2	L3	SEM
pH_0_	6.35	6.36	6.62	6.45	0.04
pH_24h_	5.46^b^	5.52^ab^	5.58^ab^	5.66^a^	0.03
Lightness (L*)	37.68	38.67	37.83	39.85	0.51
Redness (a*)	20.54	20.46	19.80	21.10	0.23
Yellowness (b*)	3.42^b^	4.19^a^	3.71^ab^	4.01^ab^	0.13
Shear force (N)	36.18^b^	28.09^c^	48.00^a^	46.74^a^	1.16
Cooking loss (%)	36.20^a^	28.29^b^	27.11^b^	27.70^b^	1.17

Different letters in peer data indicate significant difference between the groups (*P* < 0.05), same letter or no letter indicates non-significant difference between the groups (*P* > 0.05).

### Addition of Lactobacillus could affect the transformation of muscle fiber type

**FIGURE 1:**
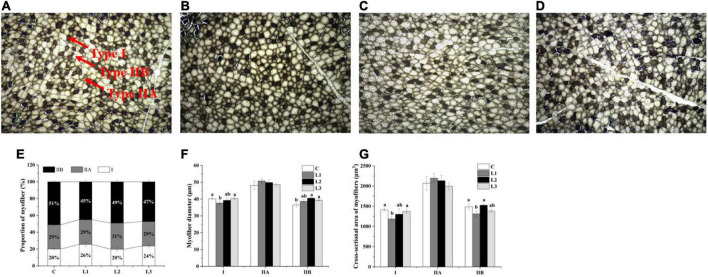
Effects of Lactobacillus on the histological properties of myofibers in Sunit sheep. **(A–D)** ATPase staining results of the C, L1, L2, L3 group. **(E)** Effects of Lactobacillus on the proportion of myofiber types. **(F)** Effects of Lactobacillus on myofiber diameter. **(G)** Effects of Lactobacillus on the cross-sectional area of myofibers. Bar: 200 μm. The myofibers observed under a 10 × 10 microscope were structurally intact, well-defined, neatly arranged, and distinctly typed. The three types I, IIA, and IIB were indicated by the arrows. Different letters indicate significant differences between the groups (*P* < 0.05), and the same or no letters indicate insignificant differences between the groups (*P* > 0.05).

**FIGURE 2:**
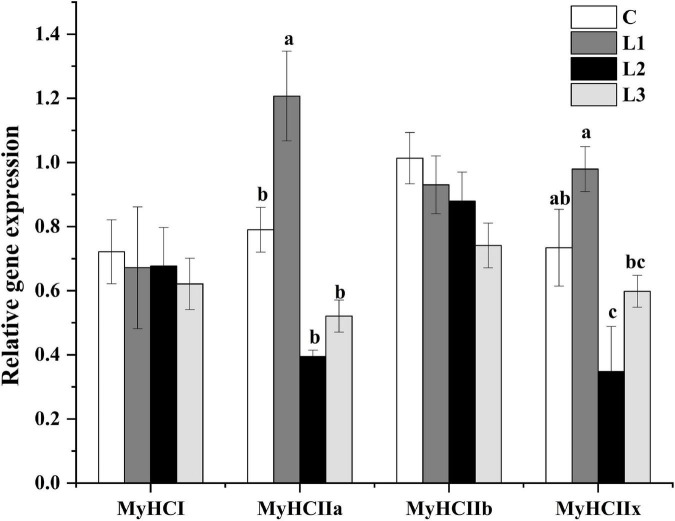
Effects of Lactobacillus on the relative gene expression of myofibers. Different letters indicate significant differences between the groups (*P* < 0.05), and the same or no letters indicate insignificant differences between the groups (*P* > 0.05).

We found that the addition of Lactobacillus could affect the histological properties of myofibers of Sunit sheep by using ATPase histochemical staining of the Sunit sheep’s femoral biceps (Figures 1A–D). The number, diameter and cross-sectional area of the different types of myofibers were statistically analyzed as described before. The myofibers of the Biceps femoris of Sunit sheep were mainly type IIB, and the oxidized type (type I and type IIA) accounted for approximately 50% (Figure 1E). As shown in Figures 1F,G, the diameter and cross-sectional area of type II myofibers were highest in all types of Sunit sheep. The cross-sectional area of type IIB myofibers in the L1 group was significantly lower than those in the C and L2 groups. Moreover, the gene expression of MyHC IIa in the L1 group was highest (Figure 2). It was shown that Lactobacillus could increase the oxidative myofiber content and decrease the glycolytic myofiber content.

### Effects of the dietary addition of Lactobacillus on metabolic enzyme activity

**FIGURE 3:**
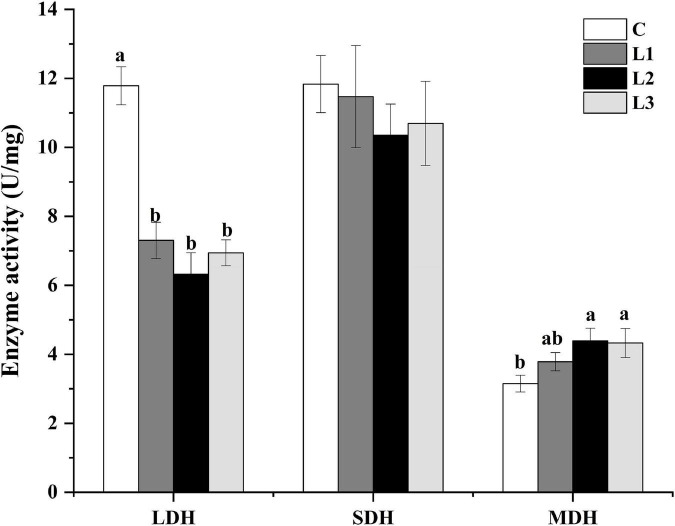
Effects of Lactobacillus on the metabolic enzyme activities of Sunit sheep. Different letters indicate significant differences between the groups (*P* < 0.05), and the same or no letters indicate insignificant differences between the groups (*P* > 0.05).

Metabolic enzyme activity can respond to changes in myofiber type. As presented in Figure 3, the LDH activity in all test groups was significantly reduced (*P*< 0.05), and the MDH activity levels in the L2 and L3 groups were significantly higher than that in the C group (*P*< 0.05). However, there was no significant difference in the activity of SDH among all the groups (*P*> 0.05). This result suggested that Lactobacillus could down regulate LDH activity, which affected muscle metabolism levels.

### Addition of Lactobacillus affects mitochondrial biogenesis through the AMPK pathway

**FIGURE 4:**
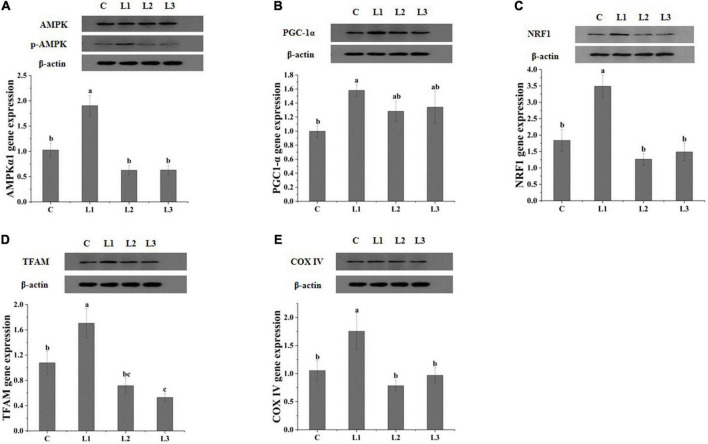
Effects of Lactobacillus on the expression of mitochondrial biogenesis-related proteins and genes in Sunit sheep. **(A–E)** Effects of Lactobacillus on the gene and protein expression of AMPK, PGC-1α, NRF1, TFAM, COX IV. Different letters indicate significant differences between the groups (*P* < 0.05), and the same or no letters indicate insignificant differences between the groups (*P* > 0.05).

To determine whether the skeletal muscle AMPK signaling pathway was activated after the dietary addition of Lactobacillus, we measured the gene and protein expression levels of AMPK, PGC-1α, NRF1, TFAM, and COX IV in the Biceps femoris of Sunit sheep, and the results are presented in Figure 4. The mRNA expression levels of AMPKα1, NFR1, TFAM, and COX IV in the L1 group were significantly higher than those in the L2, L3, and C groups (*P* < 0.05), while the mRNA expression levels of PGC-1α in the L1 group were significantly higher than those in the C group (*P* < 0.05). This finding indicated that Lactobacillus could be involved in regulating mitochondrial biogenesis via the AMPK/PGC-1α signaling pathway.

## Discussion

In this study, we reported that addition of Lactobacillus to the diet of Sunit sheep enhanced mitochondrial biogenesis in muscle by activating the AMPK pathway, which ultimately improved meat quality. Meat quality is an important factor influencing the choice of consumers which is typically evaluated by pH, tenderness, color, flavor, etc. (25, 26). It has been previously reported that modulation of myofiber type had an effect on meat quality (27). Zheng et al. (28) discovered that dietary supplementation with beta-hydroxy-beta-methyl butyrate could improve the meat quality of Bama Xiang mini-pigs through manipulation of myofiber type IIb to type I. In our study, we observed that the proportion of type I myofibers in the L1 group increased and the proportion of type IIB myofibers in the L1 group decreased, which indicated the conversion of myofibers from the glycolytic to the oxidative type. We also observed that the L1 group had the best meat quality by measuring the pH value, meat color, shear force and cooking loss. This may be related to the fact that Lactobacillus acts mainly by forming a dominant flora in the intestine. The number of viable bacteria in feeding is directly related to the effect on sheep, with too little number having no obvious effect; too much number, beyond the range of the dominant flora, may produce negative effects. Therefore, we should pay attention to the appropriate amount of use in the process of livestock breeding. It was also shown that the addition of Lactobacillus to the diet could confer good quality to meat through the conversion of myofibers from the glycolytic to the oxidative type.

Moreover, it has been reported that the conversion of myofiber type is related to metabolic enzyme activity. MDH and SDH participate in the tricarboxylic acid cycle, which can reflect the body’s aerobic metabolism level. The activities of MDH and SDH were also relatively high in muscles with more oxidative myofibers, which was in agreement with the MDH results in this experiment (29, 30). LDH is involved in glycolysis. A high LDH content will accelerate the accumulation of lactic acid in the body, affecting the quality of meat after slaughter. Xu et al. investigated the effect of proanthocyanidin B2 on myofiber type conversion and found that LDH activity was reduced during the conversion of glycolytic to oxidative myofibers, which was consistent with our results (31).

Meanwhile, the transformation of myofiber types was influenced by calcium signaling pathway, AKT-mTOR signaling pathway, AMPK signaling pathway, etc. (31–33). And it was reported that the activation of the AMPK pathway was associated with a decrease in LDH activity and increases in MDH and SDH activity (31). The AMPK pathway is a key pathway regulating mitochondrial biogenesis. In this pathway, the activation of AMPK could produce PGC-1α protein through the activation of PGC-1α, which regulates the nuclear production of NRFs and stimulates the expression of TFAM (34). It was reported that high-level expression of TFAM mRNA could increase muscle mitochondrial DNA content, increase the proportion of type I myofibers and decrease the proportion of type IIb myofibers (35). *Bifidobacterium breve* B-3 has been reported to increase the proportion of oxidative myofibers by activating the AMPK pathway by promoting high expression of PGC-1α in rat flounder muscle (36). We found that the expression of the AMPKα1 gene and the p-AMPK protein was dramatically increased in the L1 group (Figure 4A), indicating that Lactobacillus could contribute to the activation of the AMPK pathway through the phosphorylation of the AMPK protein. Moreover, the significant increases in COX IV mRNA and protein expression levels of the L1 group (Figure 4E) in our study indicated that the addition of Lactobacillus could enhance mitochondrial biogenesis by activating the AMPK pathway. COX reflects the aerobic oxidative capacity of cells, and the activity of COX IV may reflect the level of mitochondrial biogenesis (37). It has also been shown that mitochondrial biogenesis can promote skeletal muscle type transformation, which was consistent with the results of the significantly higher percentage of type I myofibers in the L1 group (Figure 1E) (38).

In summary, dietary supplementation with Lactobacillus was able to activate the AMPK signaling pathway to promote mitochondrial biogenesis and affect metabolic enzyme activity, which could regulate the transformation of glycolic myofibers to oxidative myofibers and ultimately improve meat quality. Therefore, mediating myofiber-type transformation to improve meat quality by regulating mitochondrial biogenesis is an important research direction in the future, which could promote the development of the quality meat industry. Single Lactobacillus strain selection to improve the meat quality and the related mechanisms will be involved in our further study.

## Data availability statement

The raw data supporting the conclusions of this article will be made available by the authors, without undue reservation.

## Ethics statement

The animal study was reviewed and approved by the Experimental Animal Welfare Ethics Committee of Inner Mongolia Agricultural University.

## Author contributions

YB and LSu: data collection and curation. LZ and LSn: formal analysis and software. CW and XY: visualization and writing original draft, writing review, and editing. YJ: writing review and editing. All authors have read and agreed to the published version of the manuscript.
